# Biomimetic Gradient Scaffolds Containing Hyaluronic Acid and Sr/Zn Folates for Osteochondral Tissue Engineering

**DOI:** 10.3390/polym14010012

**Published:** 2021-12-21

**Authors:** Gerardo Asensio, Lorena Benito-Garzón, Rosa Ana Ramírez-Jiménez, Yasmina Guadilla, Julian Gonzalez-Rubio, Cristina Abradelo, Juan Parra, María Rocío Martín-López, María Rosa Aguilar, Blanca Vázquez-Lasa, Luis Rojo

**Affiliations:** 1 Instituto de Ciencia y Tecnología de Polímeros, ICTP-CSIC, Calle Juan de la Cierva 3, 28006 Madrid, Spain; gerardo.asensio@ictp.csic.es (G.A.); raramirez@ictp.csic.es (R.A.R.-J.); mraguilar@ictp.csic.es (M.R.A.); bvazquez@ictp.csic.es (B.V.-L.); 2 Departamento de Anatomía e Histología Humanas, Facultad de Medicina, Universidad de Salamanca, 37007 Salamanca, Spain; lorenabenito@usal.es; 3 Departamento de Cirugía, Facultad de Medicina, Universidad de Salamanca, 37007 Salamanca, Spain; yguadilla@usal.es; 4 Departamento de Química y Bioquímica, Facultad de Farmacia, Universidad San Pablo-CEU, Urbanización Montepríncipe, CEU Universities, 28925 Alcorcon, Spain; juliangr96@hotmail.com (J.G.-R.); abradelo@ceu.es (C.A.); 5 Unidad Asociada de I+D al CSIC Unidad de Investigación Clínica y Biopatología Experimental, Complejo Asistencial de Ávila, SACYL, 05003 Avila, Spain; jparra@ictp.csic.es (J.P.); rocmar93@gmail.com (M.R.M.-L.); 6 Centro de Investigación Biomédica en Red de Bioingienería, Biomateriales y Biotecnología CIBER-BBN, Instituto de Salud Carlos III, Calle Monforte de Lemos S/N, 28029 Madrid, Spain

**Keywords:** biomimetic, tissue engineering, cryopolymerization, hyaluronic acid, folic acid, strontium, zinc

## Abstract

Regenerative therapies based on tissue engineering are becoming the most promising alternative for the treatment of osteoarthritis and rheumatoid arthritis. However, regeneration of full-thickness articular osteochondral defects that reproduces the complexity of native cartilage and osteochondral interface still remains challenging. Hence, in this work, we present the fabrication, physic-chemical characterization, and in vitro and in vivo evaluation of biomimetic hierarchical scaffolds that mimic both the spatial organization and composition of cartilage and the osteochondral interface. The scaffold is composed of a composite porous support obtained by cryopolymerization of poly(ethylene glycol) dimethacrylate (PEGDMA) in the presence of biodegradable poly(D,L-lactide-*co*-glycolide) (PLGA), bioactive tricalcium phosphate β-TCP and the bone promoting strontium folate (SrFO), with a gradient biomimetic photo-polymerized methacrylated hyaluronic acid (HAMA) based hydrogel containing the bioactive zinc folic acid derivative (ZnFO). Microscopical analysis of hierarchical scaffolds showed an open interconnected porous open microstructure and the in vitro behaviour results indicated high swelling capacity with a sustained degradation rate. In vitro release studies during 3 weeks indicated the sustained leaching of bioactive compounds, i.e., Sr^2+^, Zn^2+^ and folic acid, within a biologically active range without negative effects on human osteoblast cells (hOBs) and human articular cartilage cells (hACs) cultures. In vitro co-cultures of hOBs and hACs revealed guided cell colonization and proliferation according to the matrix microstructure and composition. In vivo rabbit-condyle experiments in a critical-sized defect model showed the ability of the biomimetic scaffold to promote the regeneration of cartilage-like tissue over the scaffold and neoformation of osteochondral tissue.

## 1. Introduction

Musculoskeletal conditions have been considered the most prevalent occupational diseases and lead to costs in medical care and social assistance with an estimated economic burden of 37 billion euros to European national health systems [1,2]. Among others, the development of musculoskeletal disorders such as osteoarthritis and rheumatoid arthritis commonly results in the progressive destruction of articular cartilage [3]. Thus, these conditions are considered a global challenge in which the development of effective regenerative therapies is crucial [4].

Current treatments for damaged articular cartilage do not represent a cure for the pathology because they only delay its progress, or they involve the creation of new defects with the possibility of increasing morbidity of the donor tissue as an adaptive immune response, as well as the creation of a focus of infections [5]. To avoid these adverse effects, regenerative therapies based on tissue engineering are becoming the most favourable and promising alternative for the regeneration of the osteochondral tissue. These new therapies combine the administration of cells, signalling molecules, and polymeric scaffolds [6].

Scaffolds must mimic the physiological properties, microstructure, and functionality of the native extracellular matrix as closely as possible to help the support of specific cellular phenotypes of each region [7,8,9]. For this reason, the design of an osteochondral regeneration scaffold needs to consider the spatial organization and composition of the osteochondral interface, which changes drastically from the highly calcified and vascularized subchondral bone zone populated by osteoblasts to the middle and superficial zones where hyaline cartilage is populated with chondrocytes and rich in collagen and glycosaminoglycans [9,10,11]. Biocompatible calcium phosphate-based materials, natural hyaluronan-based hydrogels and synthetic biodegradable polymers are generally used for the fabrication of tissue-engineered replacement of cartilage tissue [12].

Polymers of natural origin, such as hyaluronic acid (HA), have attracted great interest in the fabrication of biomimetic systems for tissue engineering alone or in combination with other natural polysaccharides [13,14,15,16,17,18] due to their biocompatibility, biodegradability, gel-forming properties and intrinsic swelling capacity [19,20]. HA is the major constituent of the synovial fluid located in the joints and has a structural function in all connective tissues [20]. Additionally, in order to increase its functionality, HA can be chemically modified by introducing methacrylate groups susceptible to participating in free-radical mediated cross-linking reactions. In this sense, covalently cross-linked structures that can swell while retaining their three-dimensional structure can be formed by photopolymerization of methacrylated hyaluronic acid (HAMA) [21,22].

Poly (D, L-lactide-*co*-glycolide) (PLGA) is a biocompatible and biodegradable polyester copolymer, which enables the growth of new tissue while it is degraded and provides rigidity and stability to the scaffold [23,24]. Furthermore, this polymer has been accepted by different health agencies such as the FDA for its use in a wide variety of therapeutic devices [25]. Poly (ethylene glycol) (PEG) is a highly hydrophilic biocompatible polymer that combined in its methacrylated form (PEGDMA) is a highly hydrophilic polymer that can be formed as a crosslinked hydrogels by means of advanced methods of manufacturing porous matrices [26,27]. Cryopolymerization is used to fabricate highly porous structures optimal for tissue engineering applications [26,27]. The preparation of cryogels involves polymerization at subzero temperatures that lead to the formation of a 3D scaffold able to promote cell adhesion, maintain the diffusion of solutes and degrade at an adequate rate [28,29]. Specifically, interconnected macropore structure allows the vascularization characteristic of the subchondral bone tissue [30] and permit the creation of stratified multiphasic, drug delivery three-dimensional scaffolds with the capacity of mimicking the microstructure and composition of the native osteochondral tissue. However, due to the intrinsic complexity of the osteochondral tissue, only a few systems have been considered for clinical trials where composite hydrogels have shown the most promising results among other polymeric systems due to their capability to form highly hydrated three-dimensional matrices from hydrophilic polymers through chemical or physical crosslinking reaction while keeping the incorporated cells or biomolecules undamaged [31,32]. One of the main challenges in obtaining multi-layered scaffolds consists in the recreation of the hieratical microstructure and smooth gradient transition between the subchondral and the cartilage mimicking layers, together of improvement of the distribution of the bioactive factors able to drive precisely and selectively the metabolism and cell-fate across the different stratus of the scaffold [33]. Thus, in order to promote adequate regeneration, it is also necessary to include bioactive agents in the system to trigger a specific cellular response targeted towards the generation of a new extracellular matrix [7,34,35,36]. In the case of bone tissue, one of the most studied agents is *β*-tricalcium phosphate (*β*-TCP), which is a bioceramic that recreates the bone microenvironment of the subchondral region [37]. In this respect, our research group previously has demonstrated the ability of new strontium and zinc folic acid derivatives (SrFO and ZnFO) to enhance the mechanisms of osteogenesis in vitro and in vivo [38,39,40], Additionally, Zn^2+^ has been described as a regulatory factor in the degradation of collagen and other components of the extracellular matrix when osteoarthritis is diagnosed [41,42].

Hence, we present the fabrication of a new bio-hybrid scaffold made of calcium phosphate-based materials, natural hyaluronan-based hydrogels and synthetic biodegradable PLGA, for osteochondral regeneration in which the combination of biomimetic hieratically gradient microstructure and presence of bioactive *β*-TCP, SrFO, ZnFO, and hyaluronic acid ingredients can drive the fate and selective colonization of human articular chondrocytes (hACs) and human osteoblast (hOBs) co-cultures within 3D scaffolds. In a step forward versus other SrFO-containing scaffolds previously reported by or group [43], the combination of a smooth gradient microstructure and the simultaneous action of ZnFO and SrFO agents in preferential zones within the same scaffold will benefit the successful regeneration of osteochondral tissues as evidenced by an in vivo critical-sized defect model on rabbit condyle.

## 2. Materials and Methods

### 2.1. Materials

PLGA (Mn = 13,600 Da, Mw/Mn = 3.7 and 50:50 lactide: glycolide RESOMER^®^ RG 502) was purchased from Evonik Industries (Darmstadt, Germany), PEGDMA (Mn = 875 Da Mw/Mn = 1) from Sigma-Aldrich and hyaluronic acid (sodium salt, M_n_ = 250 kDa, Mw/Mn = 1.6, oral grade) from Bioibérica. *β*-TCP (Merck) was triturated in an agate mortar to reduce the particles size before use. The initiators used were APS (ammonium persulfate 98%) from Sigma-Aldrich (Spain) together with TEMED (*N*, *N*,*N*’,*N*’- tetramethylethylenediamine 99.9%) from Acros Organics (Spain), and Irgacure2959 (1-[4-(2-hydroxyethoxy)-phenyl]-2-hydroxy-2-methyl-1-propane-1-one, 98%) from Sigma-Aldrich. The phosphate buffered solution (PBS: NaPO_4_ 0.01 M, 0.0027 M KCl, 0.137 M NaCl, pH 7.4) was prepared by dissolving tablets, purchased from Fisher BioReagents (Spain), in deionized water, and the remaining solvents purchased from Sigma-Aldrich and used without additional purification. Strontium and zinc folic acid derivatives were obtained as previously reported [40] with molecular formulas of SrC_19_N_7_O_10_H_25_·4H_2_O and ZnC_19_N_7_O_10_H_25_·4H_2_O, respectively.

### 2.2. Porous Support Fabrication

A mixture of dioxane/PBS (2 mL, 80:20), previously deoxygenated with a stream of nitrogen, was placed in a polypropylene tubular reactor (15 mm diameter), and PLGA (4% *w*:*v*), PEGDMA (10% *v*:*v*), *β*-TCP (1.5% *w*:*v*), SrFO (1% *w*:*v*) and APS (0.2% *w*:*v*) were added and dissolved under magnetic stirring at room temperature. Then, the magnet was then removed and TEMED (0.4% *v*:*v*) added. Next, the tubular reactor was introduced in a cryostat (Huber, model Ministat230) at −20 °C and left to react overnight. Afterwards, the tubes were thawed and the formed cryogels were washed with deionized water cut into slides of 2 mm width and punched into cylinders with a final diameter of 2.5 mm, followed by lyophilization. *β*-TCP particles size distribution was characterized in water by dynamic light scattering (DLS) using a laser diffraction particle size analyser Coulter LS230 (Beckman Electronics, Fremont, California, USA), small volume module plus, connected with a software LS32. The chemical composition of supports was analyzed by energy-dispersive X-ray spectroscopy (EDS, Bruker XFlash model with detector 5030) with a Hitachi SU8000 device, and microstructure was studied by scanning electron microscopy (SEM) using a Phillips XL30 device with tungsten filament at an acceleration voltage of 25 kV.

### 2.3. Preparation of Biomimetic Scaffolds

The synthesis of HAMA was carried out by an automatized large-scaled method using a Titrando^®^ equipment to keep a constant value of pH at 8.5. An aqueous solution of HA (5 g, 500 mL) was prepared and purged with a flux of nitrogen for 30 min before the reaction. The methacrylic anhydride (39 mL, 20 eq) was then added under magnetic stirring and the pH of the reaction automatically adjusted with NaOH 5 M up to 8.5. The reaction was left overnight, after which the pH was neutralized. Then, the reaction crude was precipitated in absolute ethanol and a white solid was formed. The product was separated by centrifugation, dialyzed against water for 3 days and lyophilized. Modification degree was determined from the ^1^H-NMR spectra recorded in a Varian INOVA-400 (at 400 and 100 MHz) taking the relative integration of the vinyl protons of the methacrylate group at 5.8–6.3 ppm (2H, s) and those of pendant acetyl protons of HA at 2.0 ppm (3H, s) giving a value of 40% (Supplementary Materials Figure S1). The molecular weight of HAMA and commercial HA was determined by size exclusion chromatography (GPC) using a Shimadzu modular system comprising: DGU-20A3 solvent degasser, LC-20AD pump, column oven, HT- autosampler 20A HT, and RID-10A refractive index detector. The samples were dissolved (2 mg/mL) in the mobile phase based on Milli-Q water with NaNO_3_ (0.2 M) and NaHPO_4_ (0.01 M). Results demonstrated that both commercial HA and functionalized HAMA presented similar molecular weight values (M_n_ = 250 kDa, Mw/Mn = 1.93) confirming the lack of side reactions.

For the preparation of the HAMA hydrogel, HAMA was weighed and dissolved at 8% (*w*:*v*) in PBS previously purged with N_2_. Irgacure2959 was initially dissolved at 5% *v*:*v* in absolute ethanol and then added to the HAMA solution at 1% regarding the weight of HAMA. After homogenizing the mixture, 25 μL were placed on the porous scaffolds letting the solution penetrate the middle of the scaffold. Next, the system was light-cured using a photoreactor (UVP, model CL-1000) equipped with five UV light bulbs with a light emission centred at 313 nm that generates a power of 0.95 W/m^2^. After 5 min, a hydrogel was formed on the top of the scaffold and 1 μL of a saturated solution of ZnFO in PBS was immediately deposited on the HAMA hydrogel.

The final biomimetic scaffolds were left to dry overnight at 40 °C. Microscopical analysis of the scaffolds in a dry and swelled state was carried out with an optical microscope Nikon Eclipse E400, and using environmental scanning electron microscopy (ESEM Phillips XL30) with a water vapour pressure comprised between 1 and 7 torrs and maintaining the sample at 3 °C.

### 2.4. Swelling and Degradation Behaviour

The swelling and degradation of prepared scaffolds were determined by gravimetric measurements after immersing the scaffolds in PBS of pH 7.4 at 37 °C. Scaffold weights and mass increments were recorded at 24 h, 72 h and 7 and 10 days. The swelling degree was related to the initial weight of the dry scaffold which defined the value of 100% and the evolution of weight loss was expressed as the percentage of weight loss, after removing the supernatant and freeze-drying the sample, in relation to the initial dry weight of the scaffolds up to a time of 10 days. All measurements were obtained with triplicate samples and results were given as mean ± standard deviation (SD).

### 2.5. Release of Sr^2+^, Zn^2+^ and Folic Acid

Biomimetic scaffolds were stored in 1.5 mL of PBS of pH 7.4 at 37 °C under stirring. After each studied time, 100 µL aliquots were taken, and the total volume was readjusted to 1.5 mL with PBS. The quantification of Zn^2+^ and Sr^2+^ was carried out by determining the content of these cations in the supernatant using a plasma-induced atomic emission spectrometer (ICP-OES) (Perkin-Elmer 430DV) registered at 408 nm for Sr^2+^ and 206 nm for Zn^2+^. Results obtained were expressed regarding the amount of SrFO/ZnFO added to the scaffolds and absorbance was recorded at 2, 4, 6, 8, 24 h, and 4 and 7 days. The amount of folic acid released from each scaffold was determined from a calibration curve obtained from the absorbance recorded at 282 nm of a series of standard solutions of folic acid in PBS with a concentration range from 2.5 mg/mL to 100 mg/mL (Abs = 0.01628Conc + 0.0224; R = 0.998). Absorption spectra were obtained with a UV/visible NanodropOne^®^ spectrophotometer. Each sample was analyzed in triplicate and the results given as mean ± SD.

### 2.6. In Vitro Biological Assays

#### 2.6.1. Cell Cultures

Cytotoxicity assay was tested on hOBs obtained from the femoral bone tissue (Innoprot, Derio, Spain), and hACs obtained from the articular cartilage of the knee (Innoprot, Derio, Spain). hOBs were cultured in osteoblast medium (Osteoblast Medium KIT, Innoprot) and hACs in chondrocyte medium (Chondrocyte Medium KIT, Innoprot) both supplemented with the respective supplier’s kit. The rest of the in vitro biological tests were carried out with a co-culture of hOBs and hACs in the biomimetic scaffold. Both lineages were co-cultured together in a chondrocyte medium supplemented chondrocyte supplier’s kit. All cell cultures were maintained under a humidified 5% CO_2_ atmosphere at 37 °C in a Thermo Fisher incubator model 3541. 

#### 2.6.2. Cytotoxicity

Cytotoxicity of biomimetic scaffolds was carried out through an indirect test following the methodology described in the ISO 10993-5:2009 [44] standard using the AlamarBlue^®^ reagent (Invitrogen, USA). Samples were placed in Eppendorf tubes with 1 mL of medium and kept at 37 °C under shaking. After 1, 2, 7 and 14 days, the extracts were collected (1 mL) and stored at −80 °C. The volume of each well was refilled with fresh medium. After 14 days, hOBs and hACs cells were seeded separately in two 96 well plates (one for each control time) at a concentration of 9 × 10^4^ cells/mL (100 μL/well) and were expanded for 24 h at 37 °C and 5% CO_2_. The next day, the medium was removed and 100 μL of the previous extract were added to each well and incubated for 24 h. Then, the extracts of the plates were removed and 100 μL of AlamarBlue^®^ reagent at 10% (v:v) in medium without phenol red were added per well, incubated for 3 h, and fluorescence was recorded at an excitation wavelength of 530/25 nm and at an emission wavelength of 590/35 nm using a Biotek Synergy HT plates (Biotek Instruments, USA). Cells incubated with a culture medium were used as a positive control. The diagrams include the mean ± SD (n = 8). ANOVA of the results of tested samples was performed with respect to control at a significance level of p < 0.001.

#### 2.6.3. Cell Adhesion and Proliferation

Cell adhesion and proliferation were studied by confocal imaging (Leica TCS SP2 confocal microscope). A co-seeding of hOBs and hACs (6 × 10^4^ cells) was performed on each scaffold sample. Both cell lineages were cultured in the chondrocyte medium described above. Before to co-seeding, hOBs nucleus and hACs cytoplasm were marked with Höechst (Höechst 33342 trihydrochloride solution, Invitrogen) and Vibrant (CFDA SE cell tracer kit, Invitrogen) staining, respectively. Briefly, the Höechst staining solution was added to hOBs cell solution at a concentration of 0.5 µg/mL, incubated at 37 °C for 15 min, followed by centrifugation and the pellet washed with PBS. After that, 7 µL of hOBs were seeded on the porous section of each scaffold and subsequently incubated at 37 °C and 5% CO_2_ for 40 min. Afterwards, 200 µL of culture medium were added and cultured for 24 h for hOBs adhesion. After this pre-culture, Vibrant probe was added at 0.5 µg/mL to hACs cell solution, and the mixture was incubated at 37 °C for 15 min in dark conditions. The solution was centrifuged, and a pellet was washed with PBS three times. After that, 7 µL of hACs were seeded on the hydrogel section of each scaffold and subsequently incubated at 37 °C and 5% CO_2_ for 40 min. Afterwards, 150 µL of culture medium were added and cultured for cell viability studies.

#### 2.6.4. Cell Viability

Adhesion and metabolic activity of the cells in the biomimetic scaffolds were determined by direct assay with the AlamarBlue^®^ reagent. After 1, 2, 7 and 14 days of culture, the scaffolds were transferred to a new 48 well plate and 500 μL of 10% (v:v) AlamarBlue^®^ solution in medium without phenol red were added to each well. After 3 h of incubation, 100 μL of AlamarBlue^®^ solution were transferred to a 96-well cell culture plate (Greiner Bio-one, Frickenhausen, Germany) in triplicate. Fluorescence was measured at an excitation wavelength of 530/25 nm and an emission wavelength of 590/35 nm using Biotek Synergy HT plates (Biotek Instruments, USA). The diagrams include the mean ± SD (n = 5). ANOVA of the results was performed comparing time points each other at significance levels of * p < 0.05, ** p < 0.005, *** p < 0.001.

#### 2.6.5. In Vitro Histological Evaluation

At 2 weeks after co-culturing hAC and hOB on the scaffolds, they were fixed in 3.7% paraformaldehyde solution for 3 h, washed with PBS, embedded in paraffin, and sectioned with a microtome (HistoCore Multicut microtome equipment, Leica Biosystems) at a thickness of 10 µm. Then, the sections were floated in a water bath at 40 °C, positioned on poly-L-lysine-coated microscope slides, baked overnight at 37 °C, stained with hematoxylin and eosin (H&E), and images recorded with a Nikon Eclipse E600 microscope with an attached Nikon DS-Fi1-L2 camera. Processing, staining, and mounting of samples were performed with a BenchMark Ultra IHC/ISH, and VENTANA HE 600 systems (Roche Tissue Diagnostics). In vitro histological evaluations were performed on the processed samples.

### 2.7. In Vivo Performance

The potential cartilage regeneration capacity of the biomimetic scaffolds was assessed in vivo in a standardized condyle model of male New Zealand White rabbits (n = 16) (18–24 months) using a rabbit osteochondral defect model. The animal experiments were carried out according to the European Directive (2010/63/EU) and the national Spanish law (RD 53/2013). Furthermore, the Ethical Committee of the University of Salamanca approved surgical protocols (register number: 423). All animals were acclimatized for at least 2 weeks prior to surgery.

Animals were pre-anaesthetized with an intramuscular injection of midazolam (2 mg/mL) and a subcutaneous dose of buprenorphine (0.05mg/kg) followed by general anaesthesia by inhalation of 1.5% isofluorane. After shaving with an electric shaver and sterilizing with 2% alcoholic chlorhexidine, an arthrotomy of the left knee joint was performed by an antero-medial parapatellar incision, dislocating the patella laterally and exposing the surface of the patellofemoral groove. Full-thickness articular osteochondral defects (4 mm in diameter and 5 mm in depth) were created in the trochlear groove of the distal femoral condyle of the joint using a surgical drill. Rabbits were randomized and divided into two groups: control (non-treated defect, n = 6) and biomimetic scaffolds (n = 10). The wounds were then carefully sewed up with a standard surgical procedure. After surgery, fentanyl patches (25 mg/h transdermally, every 72 h) and meloxicam (0.5 mg/kg, subcutaneously, every 24 h) were used for 5 days. Subcutaneous enrofloxacin (10 mg/kg every 24 h) injection was used for 5 days as antibiotic prophylaxis.

After 4- and 12-weeks post-surgery, the animals were sacrificed using an intravenous overdose injection of pentobarbital sodium. Osteochondral samples were harvested, fixed in 10% formaldehyde solution. Non-decalcified samples were embedded in plastic and processed for histological studies. Thin sections were stained with Goldner Trichrome.

## 3. Results

### 3.1. Preparation of the Porous Composite Support Scaffold

Porous composite supports were obtained by cryopolymerization of PEGDMA in the presence of PLGA, and osteoconductive agents, i.e., *β*-TCP and SrFO. The *β*-TCP particles incorporated into these supports presented bimodal particle size distribution centred at 152 and 393 μm in diameter as obtained by DLS. These particles presented adequate average sizes compared to those of the commercial ones which had an average particle diameter of 495 μm and a broad particle size distribution (Supplementary Materials Figure S3). 

**Figure 1:**
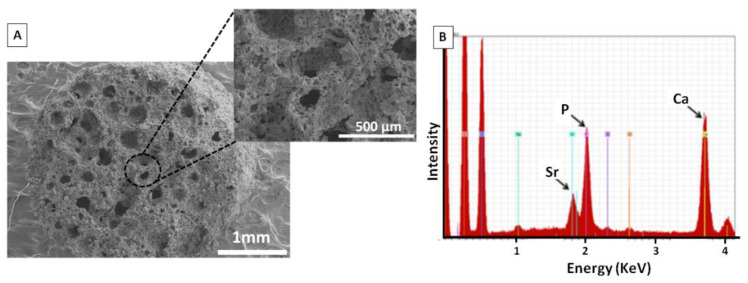
(**A**) SEM images of the porous support and (**B**) elemental surface analysis as determined by EDS.

Microstructure and chemical composition of these supports were evaluated by SEM and EDS spectroscopies, respectively (Figure 1). SEM images showed a homogeneous microstructure and a network of interconnected macro- and micro-pores. EDS analysis exhibited characteristic peaks of Sr (1.80 KeV), P (2.01 Kev), and Ca (3.69 KeV) confirming the presence of osteoconductive SrFO and *β*-TCP agents into the polymeric matrix.

### 3.2. Preparation of Biomimetic Hierarchical Scaffolds

**Figure 2:**
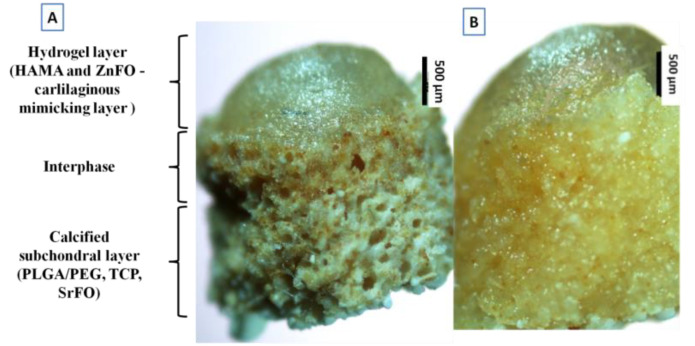
(**A**) Optical microscopy observation of the stratified microstructure of dry scaffolds (150× magnification); the porous support mimics the calcified subchondral layer, HAMA hydrogel recreates the cartilage region, and the intermediate region exhibits a combination of characteristics of regions with different composition and microstructure. (**B**) Optical microscopy of the surface gradient scaffolds after immersion in aqueous media (150× magnification).

Biomimetic scaffolds containing both synthetic and natural polymers and loaded with osteoconductive agents, i.e., β-TCP and metal folic acid derivatives were fabricated in a two-step process to obtain a hierarchical macrostructure and porous microstructure. Thus, optical microscopy pictures shown in Figure 2 confirmed the achievement of a stratified system with gradients in both composition and microstructure varying from top to bottom. Therefore, the two-step manufacturing process leads to a structure composed of three regions: a bottom highly porous zone, an upper zone of HA-based hydrogel, and a porous-hydrophilic interface which exhibited intermediate properties (Figure 2A). In addition, this hierarchical structure provided a continuous gradient microstructure with smooth transition between zones after immersion in an aqueous environment as can be observed in Figure 2B.

**Figure 3:**
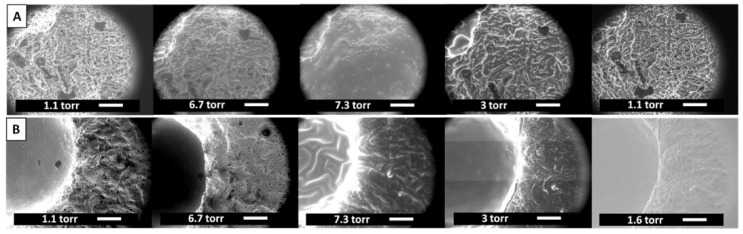
ESEM images in the wet mode of the biomimetic scaffold during hydration and dehydration cycles as controlled by water vapour pressure. (**A**) Selected top-view images showing the HAMA hydrogel (left size of the images) scale bars = 200 µm, and (**B**) Bottom-view images of the porous support. Scale bars = 100 µm.

Hydration of scaffolds was studied by ESEM microanalysis applying consecutive cycles of pressurization and depressurization in wet conditions. Images displayed in Figure 3 revealed selective hydration depending on the scaffold region. The HAMA hydrogel was capable of swelling under hydration conditions, but it reached the collapse of the structure in a dry environment (Figure 3A). However, the interconnected canals and pores, and dense polymer walls that comprised the osteoconductive support, inhibited the collapse of the structure restoring the original state in dry conditions (Figure 3B). An animated representation of the process is shown in Supplementary Materials Figure S4.

### 3.3. Swelling/Degradation Behaviour

**Figure 4:**
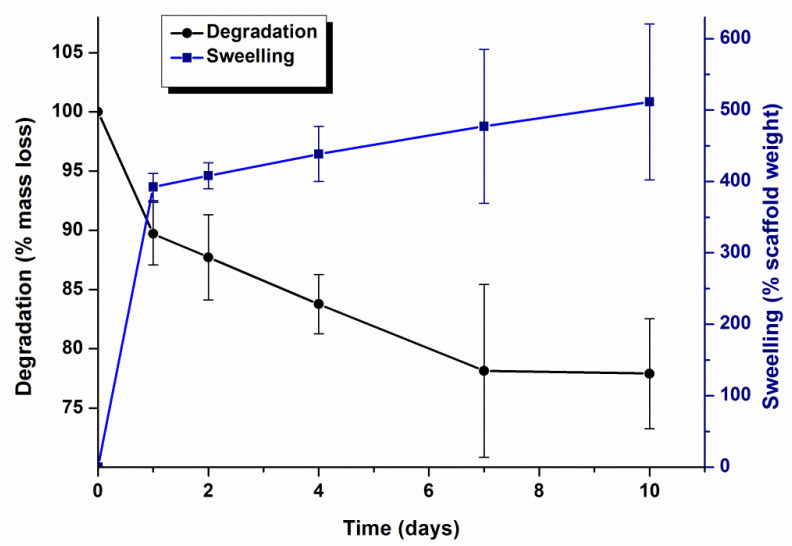
Swelling and degradation profiles of biomimetic scaffolds after 10 days of immersion in PBS of pH 7.4 at 37 °C. Lines are a guide to the eye.

Swelling capacity and degradation of biomimetic scaffolds were studied gravimetrically by immersing the samples in simulated buffered physiological conditions (PBS of pH 7.4, 37 °C) and results are shown in Figure 4. Scaffolds exhibited a high swelling capacity registering an increase of 400% in mass in the first 24 h. Afterwards, a slight and sustained increment in mass was registered reaching a 500% increment in 10 days. Likewise, a two-step degradation profile was observed in Figure 4. In the first 24 h, scaffolds experienced a decrease of 10% in mass followed by a slower degradation rate by which the remaining scaffold mass was 78% of the initial weight. 

### 3.4. Release of Sr^2+^, Zn^2+^ and Folic Acid

**Figure 5:**
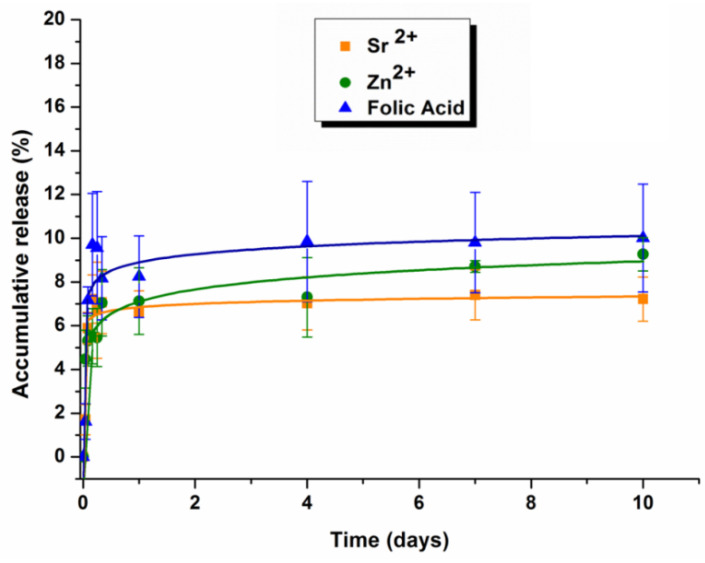
Sr^2 +^ and Zn^2 +^ and folic acid cumulative release profile from biomimetic scaffolds after 10 days of immersion in PBS of pH 7.4, at 37 °C, as determined by UV- absorbance and ICP-OES. Lines are a guide to the eye.

Release profiles of Sr^2+^, Zn^2+^ and folic acid from scaffolds are displayed in Figure 5. Two stages can be identified in them. In the first hour of the study, a burst release of bioactive compounds took place [19] delivering mainly the species that were at the surface of the scaffold. After 4 h of immersion, the concentration profile reached a plateau in which the cumulative release remained constant after 10 days of incubation. The maximum concentrations of each species released at the end of the experiment were 612.39, 87.69 and 0.44 μg/mL for folic acid, Sr^2+^, and Zn^2+^, respectively, as determined by ICP-OES analysis and by UV-vis spectroscopy.

### 3.5. In Vitro Biological Evaluation

#### 3.5.1. Cytotoxicity

**Figure 6:**
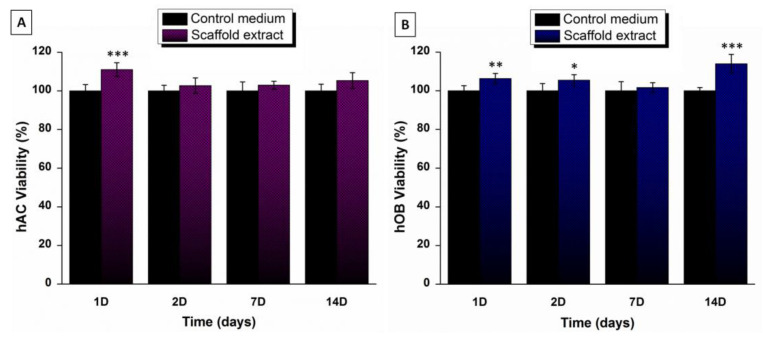
Cytotoxicity indirect assay on hACs (**A**) and hOBs (**B**) with scaffold extracts collected after 1, 2, 7 and 14 days in PBS of pH 7.4 at 37 °C. Values represent mean ± SD (n = 8). ANOVA test was performed between results of studied extracts with respect to control (basal medium) at each time point (* *p* < 0.05, ** *p* < 0.005, *** *p* < 0.001).

Cytotoxicity of lixiviates of scaffolds collected in culture medium at different time points was evaluated in hAC and hOB lines separately. Figure 6 displays the effects of the extracts on the respective cell viabilities. It was observed that cell growth of any line was not negatively affected by any extract while a significant increase in cell viability was observed at day 1 for hACs, and at days 1, 2 and 14 for hOBs. Generally, results showed the excellent biocompatibility of the system.

#### 3.5.2. Cell Viability

**Figure 7:**
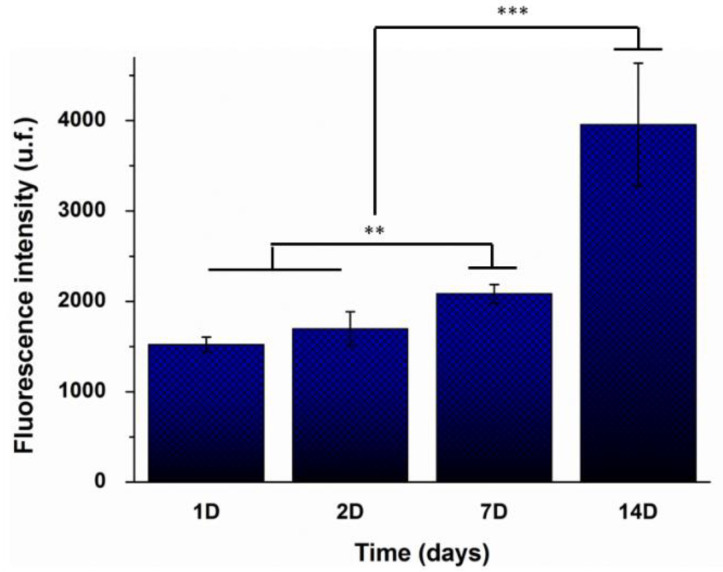
Cell viability of hACs and hOBs co-cultured on the biomimetic scaffolds after 1, 2, 7, and 14 days of incubation. Values represent mean ± SD (n = 5). ANOVA of the results was performed comparing time points each other at significance levels of ** *p* < 0.005, and *** *p* < 0.001.

The cell proliferation of a hOBs-hAC co-culture on the biomimetic scaffolds was evaluated by a direct Alamar Blue^®^ staining assay and results are represented in Figure 7. Cell proliferation increased over time, and it was significantly higher at day 14 compared to previous time points. Furthermore, after 14 days of cultured, metabolic activity was two-fold higher than at 7 days.

#### 3.5.3. Cellular Colonization and Proliferation

**Figure 8:**
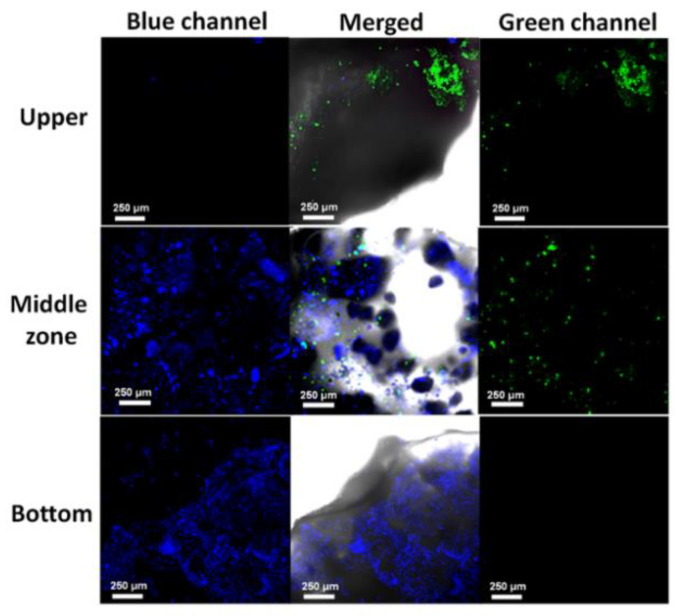
Confocal microscopy images of live stained hACs (Vibrant staining observed in the green channel) and hOBs (Höesch staining observed in the blue channel) were co-cultured on the biomimetic scaffolds after 14 days. Merge images showing the upper cartilaginous mimicking zone mainly colonized by hACs, Middle Interface zone colonized by both hOBs and hACs calcified bottom zone colonized by hOBs.

The ability of cells to colonize the scaffolds and grow inside was evaluated by confocal microscopy. Pictures displayed in Figure 8 confirmed that cells were able to colonize the biomimetic framework and successfully adhered to the polymeric matrix. Particularly, hACs were mostly found adhered to the hyaluronic acid-based hydrogel upper zone. In contrast, the intermediate region was colonized by both cell types while hOBs were distributed preferentially in the porous calcified lower zone. 

#### 3.5.4. In Vitro Histological Analysis

**Figure 9:**
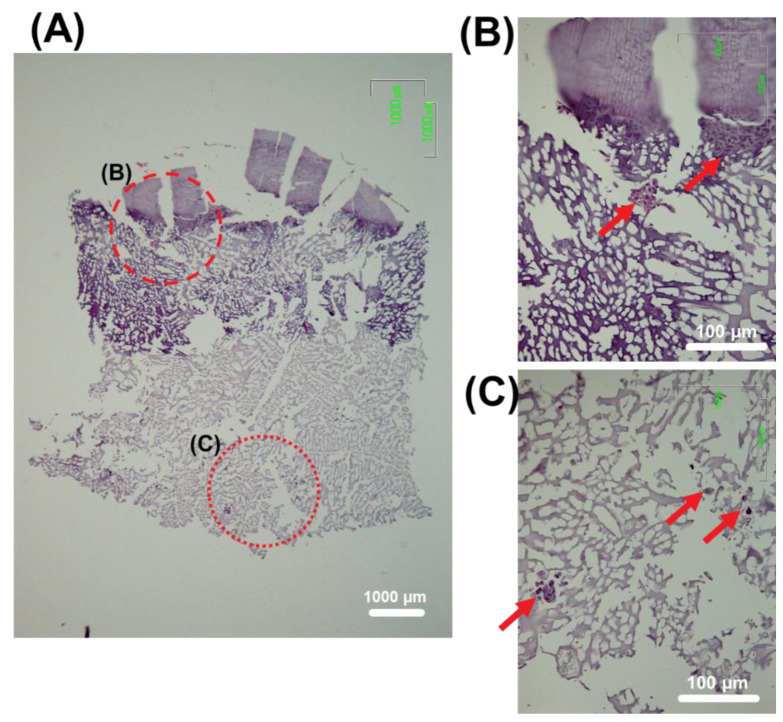
Optical microscopy images of a representative slide of the biomimetic scaffold after 14 days of seeding. (**A**): General view showing the whole piece (40×). (**B**) and (**C**): Magnification (200×) of the dashed and dotted circles, respectively. Cell aggregates are pointed with arrows. H&E staining.

Subsequent in vitro histological evaluation demonstrated the proliferation of hACs and hOBs along the scaffold after 14 days of co-culture. Figure 9A and magnifications in Figure 9B evidenced that cells colonized and grew into the interstices of the inner section of the scaffolds.

### 3.6. In Vivo Performance

**Figure 10:**
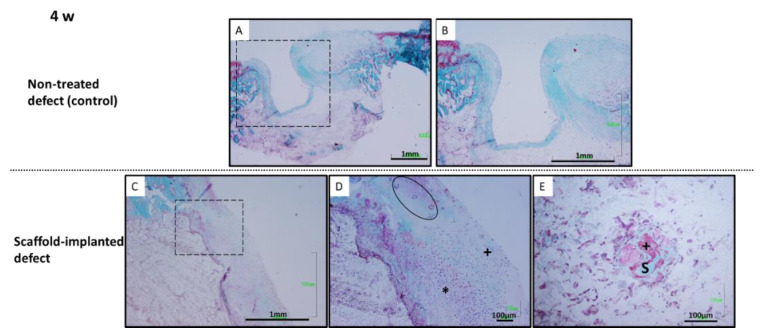
Histological evaluation at 4 weeks post-operation. Non-treated defects (**A**,**B**): fibrous tissue formation was developed at the inner part of the defect. Biomimetic scaffolds (**C**–**E**): cartilage-like tissue was regenerated, but thicker and more cellular than a normal one. Chondrocyte-like (*) and fibroblasts cells (+) were present at regenerated tissue. Chondrocytes clusters (black circle) at adjacent normal cartilage. (**E**) Isolated bone spicules (S) formation at subchondral space. Osteoid matrix (+). Goldner trichrome staining.

The in vivo response of biomimetic scaffolds was evaluated in a rabbit model by implantation of the samples into the created osteochondral defect groove. The control group had no scaffold implantation. The histological analysis was performed at 4 and 12 weeks post-implantation. At 4 weeks after implantation, the non-treated osteochondral defects presented depressed surfaces (Figure 10A) and the defects were only partially occupied by connective tissue at the inner part. There was no sign of cartilaginous tissue formation in the superficial area. The native cartilage was continued by the depressed formation of fibrous tissue (Figure 10B). However, at this time of implantation, the defects treated with the scaffolds, showed a cartilaginous tissue-like formation at the superficial part of the defects, forming similar to a bridge in continuity with the articular cartilage bordering the defect ends (Figure 10C). Chondrocyte-like cellularity could be observed in the chondral layer of the newly formed cartilage-like tissue. Cluster cellular arrangements could be appreciated at the closest zones adjacent to normal cartilage (Figure 10D). Chondrocyte-like and fibroblasts cells were present at regenerated tissue, which was thicker than native cartilage. Scaffolds allowed cartilaginous tissue formation at the upper part and avoided connective tissue development inside the defect (Figure 10C). Instead, remnants of material associated with a poor cellular reaction were detected. No accurate inflammatory reaction was appreciated associated with scaffold presence. Large residual void spaces were evident and bone repair was observed. Scarce bone spicules were formed in the subchondral space (Figure 10E).

**Figure 11:**
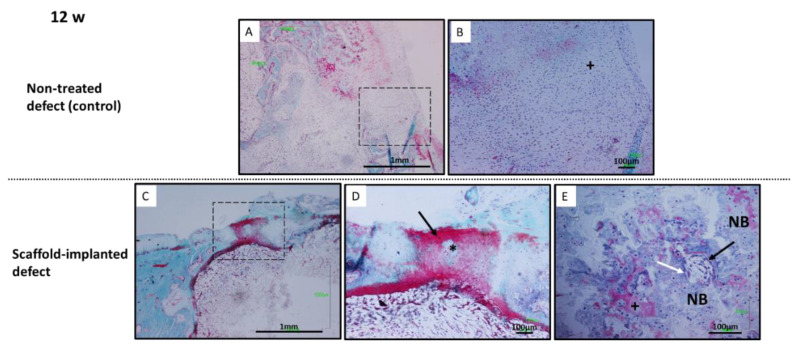
Histological evaluation at 12 weeks post-operation. Non-treated defects (**A**,**B**): the defect cavity was filled by fibrous tissue formation. Fibroblasts cells (+). Biomimetic scaffolds (**C**–**E**): (**C**,**D**) regenerated cartilage was more cellular and with more extracellular matrix compared to adjacent normal cartilage tissue. Chondrocyte-like cells (*). (**D**) Reparative tissue exhibited cartilage-like extracellular collagen matrix deposition (red staining) (arrow). (**E**) Neoformed remodelled bone was developed at subchondral space (NB). Osteoblasts (black arrow), osteoclasts (white arrow), osteoid (+). Goldner trichrome staining.

At 12 weeks post-implantation, non-treated defects were mainly occupied by fibrous tissue (Figure 11A). The connective tissue developed reached the cartilage zone formed by fibroblast-like cells and without any tissue differentiation (Figure 11B). The response of implanted scaffold defects at the upper part showed repaired cartilage-like tissue, thinner compared to 4 weeks. The regenerated cartilage developed in the defect was more cellular and with more extracellular matrix compared to adjacent normal cartilage tissue (Figure 11C). Reparative tissue exhibited cartilage-like extracellular collagen matrix deposition indicated by red staining (Figure 11D). The regenerated tissue was thicker and had irregular surfaces compared to adjacent host cartilage (Figure 11C). No gaps could be observed between the regenerated cartilage and the host cartilage, which indicated a good integration with the pre-existing tissue (Figure 11C). Neo-bone formation in the subchondral space was more evident than at 4 weeks. Discontinuous subchondral bone reconstruction was appreciated (Figure 11E). At the subchondral space scaffold material, highly vascularized loose connective tissue and bone formation could be appreciated. Neoformed bone was well remodeled and noticed osteoclasts and osteoid bone matrix deposition by osteoblasts were appreciated (Figure 11E).

No inflammatory reaction nor granulation tissue were detected in any case and at any of the experimental times, indicating good in vivo biocompatibility of studied scaffolds. Biomimetic scaffolds promoted the osteochondral healing process when compared to the non-treated group.

## 4. Discussion

### 4.1. Synthesis of Bioactive Components and Preparation of Porous Scaffolds

Despite the latest advances in tissue engineering devoted to regeneration of full-thickness articular cartilage defects, it remains challenging to repair the osteochondral interface and obtain a regenerated tissue similar to the native one. This inefficiency lies in the lack of appropriate tissue-engineered biomaterials that gather adequate composition and hierarchical structure close to those of native joint. In this context, the goal of this work was to develop biomimetic bio-hybrid hierarchical scaffolds with adequate properties to regenerate articular cartilage defects. The scaffolds were designed combining both natural and synthetic polymers loaded with bioactive cargos, in a system that mimics the complexity of the osteochondral joint. Thus, the biomimetic system presented here was obtained by a two-steps manufacturing process. The first one consisted of the preparation of bioactive porous support composed of PEGDMA/PLGA and SrFO, and *β*-TCP, which was produced by cryopolymerization. As it is known, the application of this methodology leads to the formation of a highly porous structure with interconnected channels composed of macro- and micro- pores [45] and it has been widely used in the fabrication of scaffolds for bone regeneration as they promoted osteoblasts adhesion and proliferation [29]. Accordingly, the porous framework of this work presented the referenced architecture and porosity as shown in Figure 1, with interconnected macropores (>100 μm) and micropores (<10 μm) in accordance to similar cell supports previously reported by our groups This system incorporated bioactive agents such as *β*-TCP and SrFO that endowed the matrix with osteoconductive properties [37,46,47]. The grinding process of the *β*-TCP agent allowed to obtaining the particles with average sizes closer to the values reported as optimal for bone tissue regeneration (300 μm) [48]. In addition, the resorbable capacity of *β*-TCP particles enables the formation of a new bone matrix [49,50,51]. By its part, SrFO also contributes to the osteogenic response. In a previous work, SrFO loaded PEGDMA scaffolds were evaluated in vivo in a rat calvaria animal model with induced critical size defects [39], and results demonstrated that SrFO-containing scaffolds enhanced bone regeneration compared to the control group without strontium. Moreover, the potential used of SrFO as a bone promoting factor has already been tested in vitro with human mesenchymal stem cells demonstrating their ability to modulate by their mRNA profiles towards osteogenic-like or fibrocartilaginous-like phenotypes in basal conditions [40].

The second step of the manufacturing process involved the formation of the whole biomimetic scaffold that was achieved by adding a photocrosslinked HAMA-based hydrogel onto the surface and intermediate upper part of the porous framework. Finally, the HAMA hydrogel was charged with ZnFO aiming to bust the osteochondral metabolism-enhancing subchondral bone regeneration, and also playing an important promoting cartilage II, and SOX9 gene expression and, cartilage matrix metabolism, and reduce inflammatory-related process carried out by Zn-dependent matrix metalloproteinases [52]. ESEM analysis in wet mode revealed that the as-obtained biomimetic system has spatially varying swelling properties. Scaffold pores from the porous composite bottom zone (Figure 3B) can swell as the vapour pressure of the medium increases but regain their initial shape when the pressure decreases. This means that they have an optimal open and interconnected porous structure to be colonized by osteoblasts [53,54]. However, attending to the hyaluronic acid-based zone (Figure 3A), the gel structure swells and all the pores become hydrated, but the initial state is not recovered, which implies that it is a more compact environment that resembles the cartilage matrix [9,11]. Ultimately, the fabrication procedure led to biomimetic gradient scaffolds showing a bottom-to-up heterogeneous porosity along the piece; from the bottom porous support to the top compact hydrogel part on the top of the scaffold. In the middle zone, the hyaluronic acid-based hydrogel penetrates through the porous support generating a smooth transition between zones (Figure 2 and Figure 9) and thus with a precise porous size distribution not defined.

### 4.2. In Vitro Behaviour of Biomimetic Scaffolds

Porosity properties, swelling capacity, degradation and release of soluble components must be considered collectively in tissue engineering. For the developed scaffolds, swelling and degradation phenomena takes place simultaneously (Figure 4) as reported in other cartilage engineering scaffolds [55]. Swelling kinetics showed that scaffolds can significantly increase their mass up to 400% after 24 h of immersion in physiological conditions. This initial maximum swelling can be ascribed to the most hydrophilic hyaluronic acid zone. Afterwards, the trend of the curve slows down but maintains a swelling higher than 250%. Likewise, degradation of the scaffold is initially rapid with weight loss of almost 10% in the first 24 h, and then it follows a sustained profile as has been shown in Figure 5. The first weight loss can be attributed to the release of the components, i.e., SrFO, ZnFO and folic acid, that were not embedded in the polymerized matrix, and the second region of the degradation profile can be mainly controlled by erosion processes of the polymeric matrix and resorbability of β-TCP particles. This behaviour is in accordance with other analogous polymeric matrices previously described in the literature [56,57] and, in consequence, it is expected that HA presents a higher mass-loss rate and greater swelling capacity due to its more hydrophilic character [58]. SrFO and ZnFO undergo a release of Sr^2+^, Zn^2+^, and folate ions (Figure 4) in which the three species show very similar kinetics as expected due to the coordination bond between them. In all cases, the profile begins with a burst release by which the scaffold delivers a considerable amount of the corresponding bioactive compound that was mainly adhered/close to the surface of the matrix [59]. Once this phenomenon has occurred, the release rate slows down and progresses as the degradation of the polymeric matrix takes place. This release mechanism is well accepted and it has been reported as beneficial for the extracellular environment [60]. In addition, bioactive compounds that are more rapidly released can diffuse through the extracellular fluid helping cell proliferation and matrix formation [60,61]. The presence of Zn^2+^ in the extracellular environment has been related to the activation of matrix metalloproteases responsible for the degradation of the cartilage matrix by proteolysis and is closely related to osteoarthritis mechanisms [41,62]. However, there is a therapeutic concentration range of Zn^2+^ (0.015 to 6.5 μg/mL) in which this element can have the opposite effect inhibiting the activity of these enzymes [63]. Interestingly, the concentrations of Zn^2+^ registered after the first hour of the study were high enough to exert the inhibitory function of the matrix metalloproteases without reaching toxicity. Furthermore, the concentration of Sr^2+^ found was also in the range of effective doses at all the experimental times, between 9.0 and 90.0 μg/mL [64].

### 4.3. Biological Response of Biomimetic Scaffolds

In vitro biocompatibility of the studied system was determined by indirect assay over independent cultures of hOBs and hACs. Results of cell viability in presence of extracts collected over 14 days, reveal that the processed materials do not release any toxic compound during the entire studied time, and even potential benefits can be observed for hOB cells at short and long time points, and for hACs at short time points, probably due to the effect of bioactive SrFO and ZnFO and their capability to promote osteogenic and chondrogenic processes, respectively [39,40], providing a suitable environment for cell proliferation. Confocal microscopy imaging from the different parts of the scaffold revealed the suitability of the system to sustain the colonization of hOB and hAC cells through the whole structure (Figure 9). Green staining with Vibrant for hAC cytoplasm and blue for hOB nucleus enabled to demonstrate selective hACs and/or HOBs adhesion according to the region of the scaffold that better mimics the natural extracellular matrix. The specific bind of chondrocytes to the HAMA hydrogel is regulated by the presence of CD44 in its structure [65,66]. CD44 receptors act as a link between hyaluronan-proteoglycan aggregates and the chondrocyte cell surface, and they are involved in the production of stimulatory signals that regulate chondrocytes proliferation as well as the development of new matrix [67,68]. Osteoblastic cells have exhibited the potential to adhere and proliferate onto calcium phosphate surfaces [69]. Specifically, it has been reported that *β*-TCP supports promoted adhesion of extracellular proteins that can interact with osteoblasts and exhibited high affinity to adhere to osteoblasts when compare to hydroxyapatite-based scaffolds [70,71]. Then, it is suggested that the hierarchical design obtained in this work is capable of directing in vitro cell colonization towards the different parts of the bio-hybrid scaffold according to its microstructure and composition. In addition, the existence of the middle zone with middle 3D features between the cartilage- and subchondral-mimicking layers permit the coexistence of both hOBs and hACs generating a good integration of the new forming extracellular matrix deposition, transport of nutrients and host cellular communication. The ability to mimic the smooth spatially varying properties of osteochondral tissues was also observed in vivo in a critical defect made on a rabbit condyle. The rabbit model used in this study has been previously applied in devices designed for cartilage-osteochondral repair [72]. In addition, the scaffolds do not induce any adverse reaction in the medium or long term, and despite the matrix composition (e.g., preferential Col I/Col II deposition) was not elucidated, it is proved that the scaffolds can promote the regeneration of the upper cartilage bridging the defect with a continuous cartilaginous such as tissue while inducing the formation of new bone at the subchondral zone. Thus, studied scaffolds show the ability to support host cellular infiltration and matrix deposition in vivo. Histological analysis reveals cartilaginous tissue at the superficial region of the defect site and new subchondral bone formation. Improved repair in the studied scaffold group was demonstrated in a critical size defect over the empty defect group, where fibrous tissue was developed. In summary, the biomimetic scaffold arrangement leads to tissue regeneration with a zonal organization rather similar to native osteochondral tissue.

## 5. Conclusions

In this work, we have reported the development of hierarchically structured HAMA/PLGA/PEGDMA scaffolds containing bioactive Sr/Zn folates and osteoconductive *β*-TCP, and their physic-chemical properties, and in vitro, and in vivo performance for osteochondral tissue engineering applications. The system exhibited a gradient organization in both composition and microstructure that closely recreates the physic-chemical properties of the osteochondral interface. The microstructure of the scaffolds showed its capacity to entrap and further deliver bioactive SrFO and ZnFO in biologically active concentrations. ESEM analysis revealed that each region of the scaffold had characteristic swelling ability according to the properties exhibited by the native matrix. In vitro biological results showed that the materials did not release any cytotoxic compound for hAC and hOB independent cultures, and the system allowed the proliferation of a co-cultured of both cell lineages over the studied time. Specific fluorescence staining for hOBs and hACs revealed a selective colonization trend through the scaffold structure due to its gradient composition and microstructure. Materials showed evidence of spatially varying biomimetic properties with efficient bioactive groups to emulate the osteochondral tissue components and the capacity to regenerate osteochondral critical defects in vivo by supporting the formation of cartilage-like tissue bridging the defect and inducing the formation of new bone at a subchondral level. However, it is important to highlight the need for further investigations regarding the potential of bioactive folic acid derivatives and scaffolds for clinical applications in regenerative medicine and valorize their benefits in comparison with currently available therapies.

## Data Availability

Not applicable.

## Supplementary Materials

The following are available online at https://www.mdpi.com/article/10.3390/polym14010012/s1, Figure S1: FTIR spectra of folic acid and metallic derivatives SrFO y ZnFO. Figure S2: Upper: ^1^H-NMR spectra of methacrylated hyaluronic acid (HAMA), and Lower: Chromatogram obtained of unmodified hyaluronic acid (HA) and HAMA. Figure S3: Particle size determination by Light scattering measurements of β-TCP before (grey line) and after (red line) grinder treatment. Figure S4: Animated gif (mounting of serial ESEM images obtained at different vapor pressure) representing swelling ability of top HAMA-hydrogel-based zone (Left) and bottom porous-PLGA-PEGDMA-based support (right) of biomimetic scaffold. Figure S5. Absorbance spectra of ZnFO, SrFO and Irgacure2959 (at 0.05% and 0.1% w:v).

## References

[1] Smith E., Hoy D., Cross M., Merriman T.R., Vos T., Buchbinder R., Woolf A., March L. (2014). The global burden of gout: Estimates from the Global Burden of Disease 2010 study. Ann. Rheum. Dis..

[2] Plant D., Wilson A.G., Barton A. (2014). Genetic and epigenetic predictors of responsiveness to treatment in RA. Nat. Rev. Rheumatol..

[3] Buchbinder R., Maher C., Harris I.A. (2015). Setting the research agenda for improving health care in musculoskeletal disorders. Nat. Rev. Rheumatol..

[4] (2015). European Commission Decision C H2020 WP 2014 Intro. Health, Demographic Change and Wellbeing. https://ec.europa.eu/research/participants/data/ref/h2020/wp/2014_2015/main/h2020-wp1415-health_en.pdf.

[5] Smith B.D., Grande D.A. (2015). The current state of scaffolds for musculoskeletal regenerative applications. Nat. Rev. Rheumatol..

[6] Qu H., Fu H., Han Z., Sun Y. (2019). Biomaterials for bone tissue engineering scaffolds: A review. RSC Adv..

[7] Rojo L. (2018). Combination of polymeric supports and drug delivery systems for osteochondral regeneration. Advances in Experimental Medicine and Biology.

[8] Beck E.C., Detamore M.S. (2013). Nanomaterials for Hard-Soft Tissue Interfaces.

[9] Ansari S., Khorshidi S., Karkhaneh A. (2019). Engineering of gradient osteochondral tissue: From nature to lab. Acta Biomater..

[10] Yang P.J., Temenoff J.S. (2009). Engineering Orthopedic Tissue Interfaces. Tissue Eng. Part B.

[11] Sophia Fox A.J., Bedi A., Rodeo S.A. (2009). The basic science of articular cartilage: Structure, composition, and function. Sports Health.

[12] Rahmani A., Bakhshayesh D., Asadi N., Alihemmati A., Nasrabadi H.T., Montaseri A., Davaran S., Saghati S., Akbarzadeh A., Abedelahi A. (2019). An overview of advanced biocompatible and biomimetic materials for creation of replacement structures in the musculoskeletal systems: Focusing on cartilage tissue engineering. J. Biol. Eng..

[13] Burdick J.A., Prestwich G.D. (2011). Hyaluronic Acid Hydrogels for Biomedical Applications. Adv. Mater..

[14] Highley C.B., Prestwich G.D., Burdick J.A. (2016). Recent advances in hyaluronic acid hydrogels for biomedical applications. Curr. Opin. Biotechnol..

[15] Li H., Qi Z., Zheng S., Chang Y., Kong W., Fu C., Yu Z., Yang X., Pan S. (2019). The Application of Hyaluronic Acid-Based Hydrogels in Bone and Cartilage Tissue Engineering. Adv. Mater. Sci. Eng..

[16] Kim I.L., Mauck R.L., Burdick J.A. (2011). Hydrogel design for cartilage tissue engineering: A case study with hyaluronic acid. Biomaterials.

[17] Park H., Choi B., Hu J., Lee M. (2013). Injectable chitosan hyaluronic acid hydrogels for cartilage tissue engineering. Acta Biomater..

[18] Correia C.R., Moreira-Teixeira L.S., Moroni L., Reis R.L., van Blitterswijk C.A., Karperien M., Mano J.F. (2011). Chitosan scaffolds containing hyaluronic acid for cartilage tissue engineering. Tissue Eng. Part C Methods.

[19] Pourshahrestani S., Zeimaran E., Kadri N.A., Mutlu N., Boccaccini A.R. (2020). Polymeric Hydrogel Systems as Emerging Biomaterial Platforms to Enable Hemostasis and Wound Healing. Adv. Healthc. Mater..

[20] Collins M.N., Birkinshaw C. (2013). Hyaluronic acid based scaffolds for tissue engineering—A review. Carbohydr. Polym..

[21] Laurent T.C., Bg U., Fraser J.R.E. (1996). The structure and function of hyaluronan: An overview. Immunol. Cell Biol..

[22] Park Y.D., Tirelli N., Hubbell J.A. (2003). Photopolymerized hyaluronic acid-based hydrogels and interpenetrating networks. Biomaterials.

[23] Spearman B.S., Agrawal N.K., Rubiano A., Simmons C.S., Mobini S., Schmidt C.E. (2020). Tunable methacrylated hyaluronic acid-based hydrogels as scaffolds for soft tissue engineering applications. J. Biomed. Mater. Res. Part A.

[24] Makadia H.K., Siegel S.J. (2011). Poly Lactic-co-Glycolic Acid (PLGA) as biodegradable controlled drug delivery carrier. Polymers.

[25] Loureiro J.A., Pereira M.C. (2020). PLGA based drug carrier and pharmaceutical applications: The most recent advances. Pharmaceutics.

[26] U.S. Food and Drug Administration (2017). FY2016 Regulatory Science Report: Long-Acting Injectable Formulations.

[27] Mikhalovsky S.V., Savina I.N. (2011). Biomaterials/Cryogels.

[28] Lozinsky V.I. (2002). Cryogels on the basis of natural and synthetic polymers: Preparation, properties and application. Usp. Khim..

[29] Saylan Y., Denizli A. (2019). Supermacroporous Composite Cryogels in Biomedical Applications. Gels.

[30] Hixon K.R., Lu T., Sell S.A. (2017). A comprehensive review of cryogels and their roles in tissue engineering applications. Acta Biomater..

[31] Henning M.M., van Mueller-gerbl C.N.D. (2010). The basic science of the subchondral bone. Knee.

[32] Yang J., Zhang Y.S., Yue K., Khademhosseini A. (2017). Cell-laden hydrogels for osteochondral and cartilage tissue engineering. Acta Biomater..

[33] Yan L.P., Silva-Correia J., Oliveira M.B., Vilela C., Pereira H., Sousa R.A., Mano J.F., Oliveira A.L., Oliveira J.M., Reis R.L. (2015). Bilayered silk/silk-nanoCaP scaffolds for osteochondral tissue engineering: In vitro and in vivo assessment of biological performance. Acta Biomater..

[34] Lee K., Silva E.A., Mooney D.J., Lee K., Silva E.A., Mooney D.J. (2011). Growth factor delivery-based tissue engineering: General approaches and a review of recent developments Growth factor delivery-based tissue engineering: General approaches and a review of recent developments. J. R. Cociety.

[35] Barthes J., Özçelik H., Hindié M., Ndreu-halili A., Hasan A., Vrana N.E. (2014). Cell Microenvironment Engineering and Monitoring for Tissue Engineering and Regenerative Medicine: The Recent Advances. Biomed Res. Int..

[36] Dang M., Saunders L., Niu X., Fan Y., Ma P.X. (2018). Biomimetic delivery of signals for bone tissue engineering. Bone Res..

[37] Dong J., Uemura T., Shirasaki Y., Tateishi T. (2002). Promotion of bone formation using highly pure porous b -TCP combined with bone marrow-derived osteoprogenitor cells. Biomaterials.

[38] Rojo L., Radley-Searle S., Fernandez-Gutierrez M., Rodriguez-Lorenzo L.M., Abradelo C., Deb S., San Roman J. (2015). The synthesis and characterisation of strontium and calcium folates with potential osteogenic activity. J. Mater. Chem. B.

[39] Martin-Del-Campo M., Rosales-Ibañez R., Alvarado K., Sampedro J.G., Garcia-Sepulveda C.A., Deb S., San Román J., Rojo L. (2016). Strontium folate loaded biohybrid scaffolds seeded with dental pulp stem cells induce: In vivo bone regeneration in critical sized defects. Biomater. Sci..

[40] Fernández-Villa D., Asensio G., Silva M., Ramírez-Jiménez R.A., Saldaña L., Vilaboa N., Leite-Oliveira A., San Román J., Vázquez-Lasa B., Rojo L. (2021). Vitamin B9 derivatives as carriers of bioactive cations for musculoskeletal regeneration applications: Synthesis, characterization and biological evaluation. Eur. J. Med. Chem..

[41] Troeberg L., Nagase H. (2012). Proteases involved in cartilage matrix degradation in osteoarthritis. BBA Proteins Proteom..

[42] Osorio R., Yamauti M., Osorio E., Ruiz-Requena M.E., Pashley D.H., Tay F.R., Toledano M. (2011). Zinc reduces collagen degradation in demineralized human dentin explants. J. Dent..

[43] Martín-Del-Campo M., Sampedro J.G., Flores-Cedillo M.L., Rosales-Ibañez R., Rojo L. (2019). Bone regeneration induced by strontium folate loaded biohybrid scaffolds. Molecules.

[44] International Organization Standardization, ISO 10993-5 (2009). Biological Evaluation of Medical Devices—Part 5: Tests for In Vitro Cytotoxicity.

[45] Memic A., Colombani T., Eggermont L.J., Rezaeeyazdi M., Steingold J., Rogers Z.J., Navare K.J., Mohammed H.S., Bencherif S.A. (2019). Latest Advances in Cryogel Technology for Biomedical Applications. Adv. Ther..

[46] Bohner M., Le B., Santoni G., Döbelin N. (2020). β-Tricalcium Phosphate for Bone Substitution: Synthesis and Properties. Acta Biomater..

[47] Legeros R.Z. (2008). Calcium Phosphate-Based Osteoinductive Materials. Am. Chem. Soc..

[48] Watanabe S., Takabatake K., Tsujigiwa H., Watanabe T., Tokuyama E., Ito S., Nagatsuka H., Kimata Y. (2016). Efficacy of Honeycomb TCP-induced Microenvironment on Bone Tissue Regeneration in Craniofacial Area. Int. J. Med. Sci..

[49] Ogose A., Hotta T., Kawashima H., Kondo N., Gu W., Kamura T., Endo N. (2004). Comparison of Hydroxyapatite and Beta Tricalcium Phosphate as Bone Substitutes After Excision of Bone Tumors. J. Biomed. Mater. Res. Part B Appl. Biomater..

[50] Renooij W.I.L.L.E.M., Hoogendoorn H.A., Visser W.J., Lentferink R.H., Schmitz M.G., Van Ieperen H.A.N.S., Oldenburg S.J., Janssen W.M., Akkermans L.M., Wittebol P.A.U.L. (1984). Bioresorption of Ceramic Strontium-8 5-Labeled Calcium Phosphate Implants in Dog Femora A Pilot Study to Quantitate Bioresorption of Ceramic Implants of. Clin. Orthop. Relat. Res..

[51] McAndrew M.P., Gorman P.W., Lange T.A. (1988). Tricalcium phosphate as a bone graft substitute in trauma: Preliminary report. J. Orthop. Trauma.

[52] Jiménez M., Abradelo C., San Román J., Rojo L. (2019). Bibliographic review on the state of the art of strontium and zinc based regenerative therapies. Recent developments and clinical applications. J. Mater. Chem. B.

[53] Bakhshpour M., Idil N. (2019). Biomedical Applications of Polymeric Cryogels. Appl. Sci..

[54] Vandrovcová M., Áková L.B.A.Č. (2011). Adhesion, Growth and Differentiation of Osteoblasts on Surface-Modified Materials Developed for Bone Implants. Physiol. Res..

[55] García-Fernández L., Olmeda-Lozano M., Benito-Garzón L., Pérez-Caballer A., San Román J., Vázquez-Lasa B. (2020). Injectable hydrogel-based drug delivery system for cartilage regeneration. Mater. Sci. Eng. C.

[56] Chung C., Beecham M., Mauck R.L., Burdick J.A. (2009). The influence of degradation characteristics of hyaluronic acid hydrogels on in vitro neocartilage formation by mesenchymal stem cells. Biomaterials.

[57] Fomby P., Cherlin A.J., Hadjizadeh A., Doillon C.J., Sueblinvong V., Weiss D.J., Bates J.H.T., Gilbert T., Liles W.C., Lutzko C. (2010). Effect of pore sizes of PLGA scaffolds on mechanical properties and cell behaviour for nucleus pulposus regeneration in vivo. Ann. Am. Thorac. Soc..

[58] Credi C., Biella S., de Marco C., Levi M., Suriano R., Turri S. (2014). Fine tuning and measurement of mechanical properties of crosslinked hyaluronic acid hydrogels as biomimetic scaffold coating in regenerative medicine. J. Mech. Behav. Biomed. Mater..

[59] Huang X., Brazel C.S. (2001). On the importance and mechanisms of burst release in matrix-controlled drug delivery systems. J. Control. Release.

[60] Porta G.D., Nguyen B.B., Campardelli R., Reverchon E., Fisher J.P. (2014). Synergistic effect of sustained release of growth factors and dynamic culture on osteoblastic differentiation of mesenchymal stem cells. Soc. Biomater..

[61] Zhang Y., Yu T., Peng L., Sun Q., Wei Y., Han B., Wang J., Wang D. (2020). Advancements in Hydrogel-Based Drug Sustained Release Systems for Bone Tissue Engineering. Front. Pharmacol..

[62] Nosrati R., Kheirouri S., Ghodsi R., Ojaghi H. (2019). The effects of zinc treatment on matrix metalloproteinases: A systematic review. J. Trace Elem. Med. Biol..

[63] Johnson A.R., Pavlovsky A.G., Ortwine D.F., Prior F., Man C., Bornemeier D.A., Banotai C.A., Mueller W.T., Mcconnell P., Yan C. (2007). Discovery and Characterization of a Novel Inhibitor of Matrix Metalloprotease-13 That Reduces Cartilage Damage in Vivo without Joint Fibroplasia Side Effects. J. Biol. Chem..

[64] Place E.S., Rojo L., Gentleman E., Sardinha J.P., Stevens M.M. (2011). Strontium- and Zinc-Alginate Hydrogels for Bone Tissue Engineering 1,2. Tissue Eng. Part A.

[65] Butchers E.C., Carter W.G. (1989). Structural homology between lymphocyte receptors for high endothelium and class III extracellular matrix receptor. Immunology.

[66] Radotra B., Mccormick D., Crockard A., Hospital R.V. (1994). CD44 plays a role in adhesive interactions between glioma cells and extracellular matrix components. Neuropathol. Arid Appl. Neurobiol..

[67] Ishida O., Tanaka Y., Morimoto I., Takigawa M. (1997). Chondrocytes Are Regulated by Cellular Adhesion Through CD44 and Hyaluronic Acid Pathway. J. Bone Miner. Res..

[68] Knudson C.B., Knudson W. (2004). Hyaluronan and CD44. Clin. Orthop. Relat. Res..

[69] Jeong J., Kim J.H., Shim J.H., Hwang N.S., Heo C.Y. (2019). Bioactive calcium phosphate materials and applications in bone regeneration. Biomater. Res..

[70] Euler A., Farina M., Soares G.A. (2007). Specific proliferation rates of human osteoblasts on calcium phosphate surfaces with variable concentrations of α -TCP. Mater. Sci. Eng. C.

[71] Suzuki T., Hukkanen M., Ohashi R.Y.O., Yokogawa Y. (2000). Growth and Adhesion of Osteoblast-Like Cells Derived from Neonatal Rat Calvaria on Calcium Phosphate Ceramics. J. Biosci. Bioeng..

[72] American Society for Testing and Materials (2010). ASTM F2451-05(2010), Standard Guide for in vivo Assessment of Implantable Devices Intended to Repair or Regenerate Articular Cartilage.

