# Supplementary material for: Biomimetic Gradient Scaffolds Containing Hyaluronic Acid and Sr/Zn Folates for Osteochondral Tissue Engineering
Source: Polymers (Basel). 2021 Dec 21;14(1):12. doi: 10.3390/polym14010012 (PMC8747647; doi:10.3390/polym14010012)
Supplement: Supplementary file 1 [file polymers-14-00012-s001.zip › polymers-1493598-supplementary.pdf]

Biomimetic Gradient Scaffolds Containing Hyaluronic Acid and Sr/Zn Folates for Osteochondral Tissue Engineering

Gerardo Asensio, Lorena Benito-Garzón, Rosa Ana Ramírez, Yasmina Guadilla, J. Gonzaález-Rubio, Cristina Abradelo, Juan Parra, Rocío Martín-López, María Rosa Aguilar, Blanca Vázquez-Lasa and Luis Rojo

Supplementary data

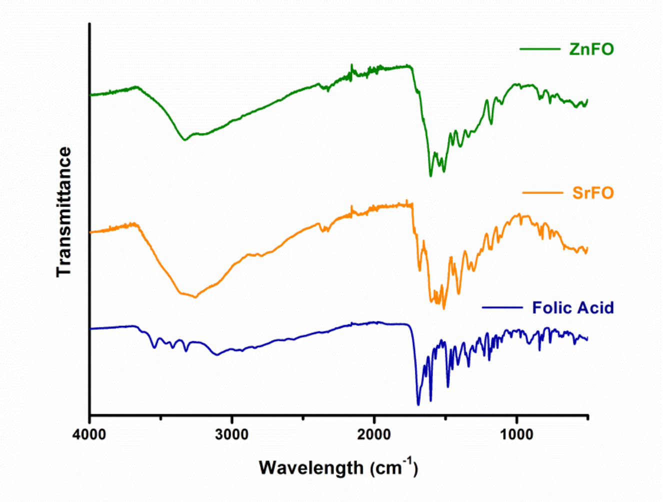

Figure S1. FTIR spectra of folic acid and metallic derivatives SrFO y ZnFO.

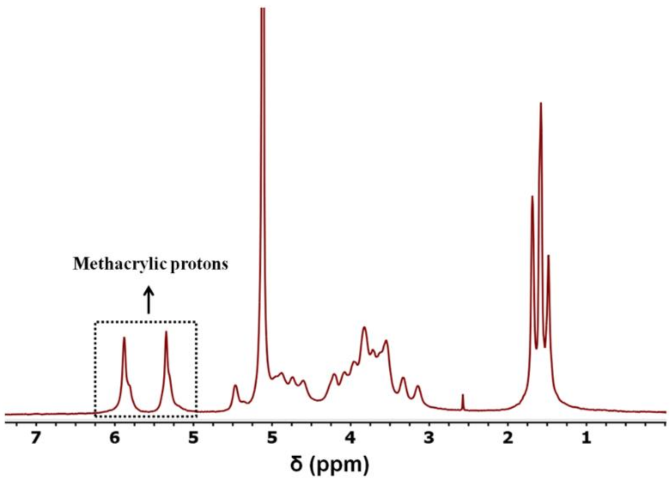

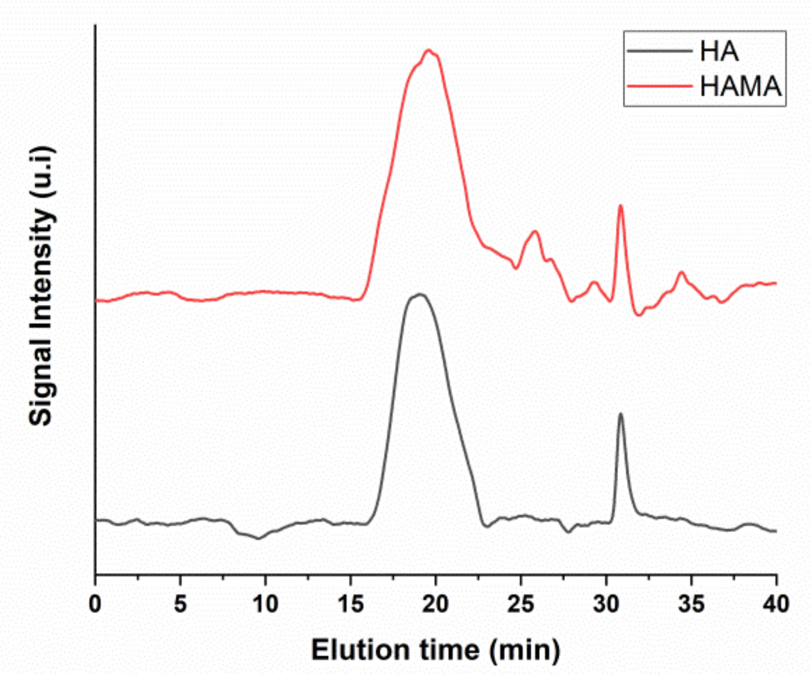

Figure S2. Upper: ^{1}H-NMR spectra of methacrylated hyaluronic acid (HAMA), and Lower: Chromatogram obtained of unmodified hyaluronic acid (HA) and HAMA .

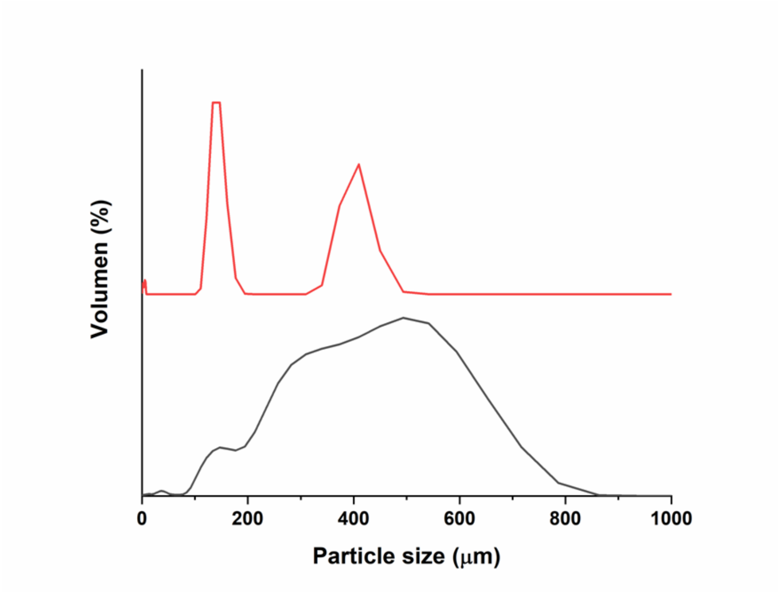

Figure S3. Particle size determination by Light scattering measurements of β-TCP before (grey line) and after (red line) grinder treatment.

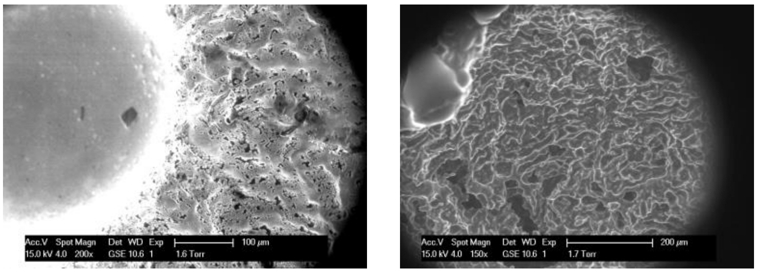

Figure S4. Animated gif (mounting of serial ESEM images obtained at different vapor pressure) representing swelling ability of top HAMA-hydrogel-based zone (Left) and bottom porous-PLGA-PEGDMA-based support (right) of biomimetic scaffold.

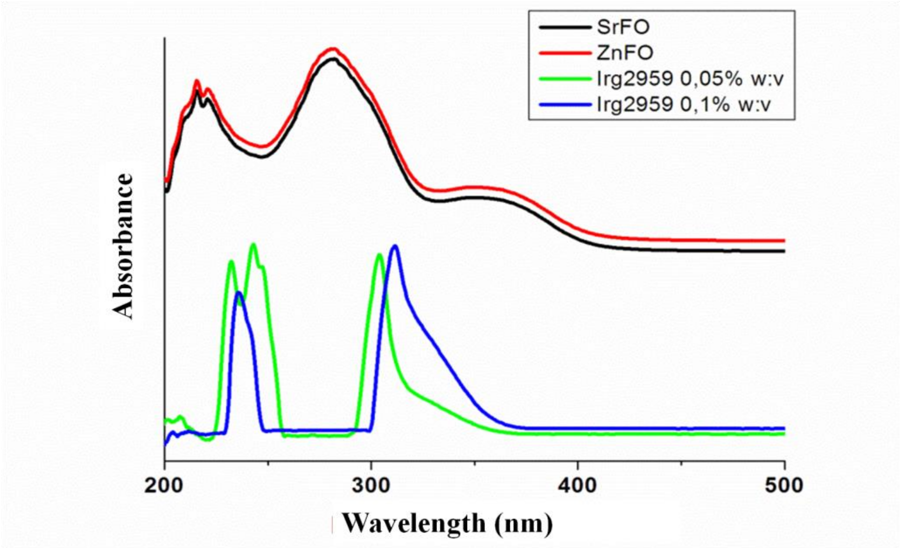

Figure S5. Absorbance spectra of ZnFO, SrFO and Irgacure2959 (at 0.05 % and 0.1 % w:v).
